# How Should We Help Wild Animals Cope with Climate Change? The Case of the Iberian Lynx

**DOI:** 10.3390/ani13030453

**Published:** 2023-01-28

**Authors:** Falco van Hassel, Bernice Bovenkerk

**Affiliations:** Philosophy Group, Social Sciences Group, Department of Communication, Philosophy, and Technology (CPT), Wageningen University and Research, Hollandseweg 1, 6706 KN Wageningen, The Netherlands

**Keywords:** Iberian lynx, climate justice, conservation, assisted migration

## Abstract

**Simple Summary:**

Many animals stand to suffer from climate change and related shifts in weather conditions, for example, because their habitat is no longer suitable and the incidence and severity of natural disasters will increase. Additionally, many species face extinction. This article explores the measures that should be taken to alleviate this suffering and prevent extinction. The Iberian lynx is taken as a case study. This species faces extinction as a result of climate change, because its habitat is becoming too dry and its main food source—the rabbit—is declining. It is argued that doing nothing or bringing the Iberian lynx into captivity are not appropriate measures. Providing food and shelter or reducing other threats (such as roads) can help but will not be enough to save the species in time. Assisted migration may be the best way to save the species and ensure animal welfare.

**Abstract:**

Climate change and related shifts in weather conditions result in massive biodiversity declines and severe animal suffering. This article explores the measures that can be taken to decrease animal suffering and prevent species from going extinct. Taking the Iberian lynx as a case study, we assess the extent to which it is beneficial for animal welfare and species conservation to do nothing or reduce other threats, provide food or shelter, relocate the species via assisted migration, or bring the population into captivity. We argue that, given the Iberian lynx’s non-invasive characteristics, assisted migration may be the best way to protect the species while ensuring animal welfare and protecting wildness and other ecosystem values.

## 1. Introduction

Anthropogenic greenhouse gas emissions are increasing global temperatures, with expectations that temperatures will continue to rise between 2 and 3 °C above pre-industrial levels by the end of the 21st century [1,2]. This temperature rise will result in drastic changes in the Earth’s system, impacting both human and non-human animals. Climate change experts and biologists predict that up to half of threatened terrestrial mammals and one-quarter of threatened birds have already been negatively affected by climate change and that 1 in 20 species is at risk of extinction as a result of climate change at 2 °C warming, increasing to 1 in 6 species at 4.3 °C global warming [3,4], resulting not only in massive biodiversity losses, but also in the suffering of unimaginably large numbers of individuals. Even if our mitigation efforts prove successful, for many species, this will not be fast enough to prevent suffering and extinction, meaning that to avoid such suffering and extinction, some form of assistance will be necessary.

Climate ethicists argue that those most vulnerable to climate change should be helped by those who caused it and are least affected by it, as summarized in the term climate justice [5]. Wealthy countries are the main drivers of climate change, whereas people in poor countries are generally the most affected. However, with our anthropocentrically focused ethics, until now, animals have been mostly excluded from this term. Yet, as argued by Pepper [6], Tschakert et al. [7], and Blattner and Meijer [8], there are good reasons to extend the scope of climate justice by including other animals besides humans. Non-human animals have not contributed significantly to the current climate change, and certainly not intentionally. At the same time, as mentioned, many species and individual animals will be adversely affected. On the assumption that climate justice can be extended to animals and that, therefore, we have a moral duty to help wild animals.(We understand wildness as a gradual notion: the more animals are dependent on humans and the more they are adapted to living in proximity to humans, the more domesticated and the less wild they are. See [9]. Palmer [10] uses a different typology of wildness, to which we shall come back later. cope with the adverse effects of climate change, what actions are warranted? In our view, this question can best be answered by examining the particulars of specific contexts. Therefore, we examine one particular case study, that of the Iberian lynx (*Lynx pardinus*). Although the particular context matters, we believe insights from this case study may be generalizable to other cases, as similar issues may be at play in other contexts. We first introduce the Iberian lynx as a case where climate justice is relevant and, moreover, where considerations of animal welfare play a role. Then we examine the moral implications of five different intervention strategies, ranging from low to high levels of interference: doing nothing, reducing other threats, providing food and shelter, assisted migration, and bringing lynx populations into captivity. Recently, researchers and philosophers have been looking into and debating the possibility of facilitated adaptation, by which animals can be genetically ‘redesigned’ to cope for example with environmental changes, e.g., by increasing the species’ drought resilience (see for example [11]). However, to date, this is not possible. In the case of the Iberian lynx, decision making is needed relatively quickly—the species may be extinct in 50 years. Even if facilitated adaptation were ethically reasonable, there is no time for the lynx to wait for a measure like this. Therefore, this paper does not investigate its moral considerations. It should be noted that potential measures to help animals cope with the effects of climate change are in no way a replacement for the mitigation measures and goals that are so important. Without an adequate mitigation strategy, the Earth’s system will continue to change and, in many situations, each measure on its own might not be enough to guarantee the species’ survival or prevent individual animals’ suffering.

In our exploration of the case study, we touch on several current discussions relating to animal and environmental ethics. First of all, the two dominant animal ethics approaches—the utilitarian and the deontological approach—differ with respect to the goals that they aim to achieve. Utilitarian ways of reasoning focus only on the outcomes of intended actions, and the utilitarian maxim is to choose the action that maximizes pleasure or happiness and minimizes pain or unhappiness for all involved. Deontological ways of reasoning do not focus primarily on outcomes, but base the morality of an action on a set of moral principles. According to the former, only the outcomes matter, whereas, according to the latter, intentions are also morally relevant. The deontological animal rights view as defended by Tom Regan [12] states that all beings that can experience their life subjectively have inherent rights that should be respected. As respect for animal rights often entails protecting animals from harm and suffering, the two positions often come to the same conclusion in practice. However, according to the rights view, certain actions (such as taking away animals’ freedom) are unacceptable in principle, whereas in the utilitarian view they may be allowed if they lead to the best outcome overall in terms of welfare or happiness. As we shall see, one issue on which they differ is whether it is morally acceptable to harm a number of individuals (for example by breeding them in captivity) in order to save a larger number [13]. One issue about which animal ethicists disagree among themselves (irrespective of whether they are of the utilitarian or the deontological persuasion) is whether the alleviation of wild animal suffering—for example, by stopping predators from killing prey—is mandatory across the board, or only when the suffering is caused by humans [14].

Moreover, as will become clear in our discussion, there is an underlying value conflict between animal ethicists on the one hand, who focus on the welfare and/or rights of individual animals, and ecocentrists on the other hand, who focus on the preservation of species and/or the value of wildness. According to many animal ethicists, species do not have moral status. In their eyes, species are made up of individual animals and these are the sole locus of moral concern; after all, species cannot have interests, only individual sentient organisms can [15,16]. Moreover, a species is a category devised by humans to classify different groups of organisms; this in itself does not make them entities with independent moral status. Ecocentrists, for their part, argue that, if we did not attach independent moral value to species, we could not explain why it is important to us to preserve species, or why, if we had to choose between saving a member of an endangered species and a member of an abundant species, we should favor the endangered one [17]. This is an ongoing and seemingly unresolved discussion (see Ref. [18]). However, as we aim to show, we think that this opposition in practice is not as insurmountable as it seems at first sight and a balance can be struck between the two approaches by combining a variety of intervention strategies. Even though our point of departure in climate justice suggests that we reason from a rights framework, we in fact look at our case study from three different perspectives: a perspective that focuses on individual animal welfare—which is more akin to a utilitarian framework—a perspective that focuses on justice for individual animals, and a perspective that focuses on species justice—the latter two being more akin to the deontological rights framework. We are aware that the question of whether species can be owed justice is a contentious one, but here we assume that it is possible. It is not our aim to take a stand on which perspective is most compelling; rather, we look for convergence between different moral perspectives in practice.

## 2. The Iberian Lynx

As climate change affects both human and non-human animals—billions of individuals worldwide—it is likely that some prioritizations need to be made when deciding who to help. If we look at this from an animal welfare perspective, we should prevent the most suffering. It has been argued that we should, therefore, focus first on the individuals who stand to suffer the most [5]. Animals affected by anthropogenically enhanced natural disasters—increased droughts, floods, and forest fires—are likely to suffer a lot. For this reason, it may be most efficient to identify areas at risk of transforming drastically and individuals of species that have difficulty adapting to such transformations. It should be noted here that we discuss our duties toward animal suffering as a result of our actions. In the animal ethics literature, a vibrant discussion is taking place about the question of whether we have a duty to try to stop wild animal suffering in general [19,20]. We do not have enough space here to elaborate on this discussion; as explained later, we follow Palmer’s argument that we have different duties toward wild than toward domesticated animals, and hence we do not hold that we have a duty to alleviate all wild animal suffering [10]. Besides prioritizing individuals of species that are most vulnerable to the effects of climate change, in order to prevent extinctions, vulnerable species should be prioritized. These are likely to be species that adapt to change slowly and have small ecological niches (non-generalists) and species that cannot move themselves to areas with characteristics similar to those in their historical range, for example, because of fragmented habitats.

We have decided to focus on the Iberian lynx, firstly because this species is at high risk of extinction and suffering [21], meaning that there are both species-based and individual-based reasons for concern. Secondly, we have selected the Iberian lynx because there is already an ongoing conservation practice in place for this species. Thirdly, the causes of the problems faced by the lynx, both in the past and in the present, are anthropogenic. This lynx species—as its name indicates—lives in the Iberian Peninsula and was near extinction a few decades ago. After rapid declines in lynx populations as a consequence of hunting, fragmentation of their habitat, and declines in the European rabbit—its main food source—measures (habitat protection and captive breeding and release) were successfully taken to increase the number of lynxes living in the wild [22]. Currently, the population is at relatively stable levels. However, scientists predict that, within 35 years, the current habitats of the lynx (in Spain) will have declined because of changing weather conditions due to climate shifts, resulting in increased droughts in the Iberian Peninsula and consequently the species’ extinction within 50 years, even with a far-reaching greenhouse gas mitigation policy [22]. The lynx is dependent on the Mediterranean shrubland and high rabbit populations, both of which are threatened by the shifts in weather condition [21,23]. Furthermore, its adaptive capacity is poor because of its low genetic diversity and fragmented populations [24,25,26]. Without human constraints, the Iberian lynx would probably have been able to survive by itself, even with the current rapid rates of climate change. It is expected that new areas suitable for the lynx will arise in Western France, to where they would have been able to migrate. Unfortunately, urbanization, fragmentation of nature areas, and a lack of wildlife corridors have led to a situation in which the lynx is limited to its current dispersal range, resulting in the species’ extinction without human intervention [21,22]. Moreover, given its poor genetic composition in the wild and its small population size, non-generalist characteristics, and low reproduction rates, the species can adapt only slowly to changes. Because of these characteristics, individual lynxes are also at high risk of suffering from increased droughts, making the species a justified candidate for human intervention.

## 3. Doing Nothing

Until recently, doing less was likely to have been the most successful measure to prevent species extinctions. Less hunting, fishing, habitat destruction, and pollution, conserving and preserving what is there, while minimizing exploitation, would have prevented many species from going extinct [27]. However, because of climate change, which causes rapid, large-scale environmental changes, new approaches may need to be adopted. The traditional doing-less preservation approach may at some point not be enough to prevent species’ extinction. As Minteer and Collins [27] contend, we may need to adopt a post-preservationist conservation, where we move from preservation to active intervention. Still, it is important to decide whether or not we should intervene in each specific case. According to Delon and Purves [28] (p. 3), ‘an intervention is morally justified only if we are sufficiently confident that the intervention will not make matters worse’. Therefore, we first examine the ethical consequences of doing nothing before we discuss the different interventions. Of course, there is a substantial risk that we will do nothing, not because of well-considered reasons, but because of indecisiveness, a lack of interest, or high levels of complexity and uncertainty regarding the case at hand. However, doing nothing does not have to be an indecision or a postponement of decision making; it can also be an active decision. Regardless of whether the decision to do nothing is made actively or not, it comes with moral consequences, as the (in)decision will impact the lynx on both the individual animal and species level.

One reason for deciding to do nothing is because we think that we are not morally obliged to do so. Palmer [29] argues that, as we have different relationships with wild as compared with domesticated animals, we have different obligations when domesticated rather than wild animals are suffering. Because we have determined the position in which domesticated animals find themselves—we determine where and how they live, what they eat, with whom they socialize or mate, we often even determine their genetic constitution—and thereby have put them in a vulnerable or at least a dependent position, we have positive duties of care toward these animals. Toward animals whose situation we have not determined we have only negative duties, meaning that we should employ a laissez-faire attitude toward them, although we do have the duty not to kill them or destroy their environment unnecessarily. Yet, as Palmer herself acknowledges, in the case of climate change, it is difficult to maintain that we have only a negative duty toward wild animals, given that we have made these animals more vulnerable by destroying their habitat and changing the climatic conditions that they face. It seems, therefore, that we have at least a *prima facie* duty to intervene in the situation of the Iberian lynx.

**Figure 1:**
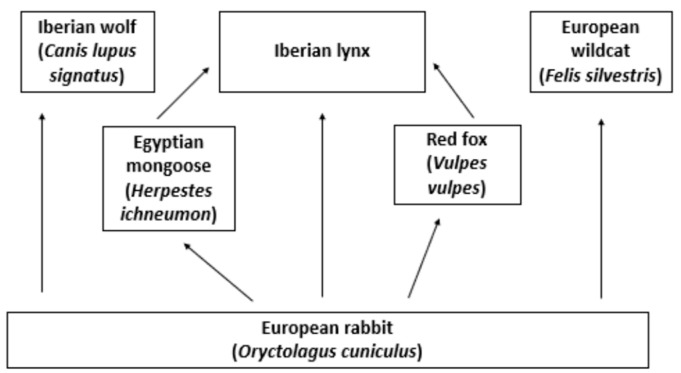
Simplified (and partial) overview of the Iberian lynx food web (based on [33,34]).

On second thoughts, however, this may not be so clear. After all, we have to take into account that intervening in ecosystems by helping certain animals often comes at the expense of other animals. If our interference in the lives of wild animals creates an obligation on our part to help these animals, as Palmer argues, this may entail ripple effects of our interferences on other than the target animal. Before intervening, we should therefore investigate the lynx’s relationships with other animals in the wild. For illustrative purposes, Figure 1 shows a simplified overview of the Iberian lynx’s food web. According to Palmer’s approach, we have moral responsibilities toward individual Iberian lynxes, as they have human-induced increased vulnerability resulting from climate change among other things. The same applies for individuals of, for example, the Iberian wolf (*Canis lupus signatus*), with a vulnerable IUCN conservation status consequent to persecution and loss of habitat [30]. Additionally, individuals of non-vulnerable species, such as the European wildcat (*Felis silvestris*), have increased vulnerability to suffering from human causes [31]. Helping individuals of one species, such as the Iberian lynx, increases the pressure on prey species and consequently is likely to increase competition between individuals of different predator species, increasing the human-induced vulnerability of individuals of the species that are not helped, to which we may then also have moral obligations. Increased Iberian lynx populations may, for example, impact the European wildcat populations significantly, as both species have rabbits as their main source of prey [32].

It could be that helping the Iberian lynx will not result in increased competition between predators given the robustness of the rabbit population, e.g., because of their high reproduction rate. Even then, however, helping individuals of one species will increase the vulnerability of individuals of another species, to which we consequently have moral obligations as well. Most definitely, individuals of the European rabbit species will be confronted with increased predation, and, with this, increased suffering compared with maintaining the status quo.

In other words, prioritizing certain individuals will increase moral obligations to other individuals negatively impacted by this prioritization. Nevertheless, it is an open question how far our moral obligations extend. As argued by Swart and Keulartz [9], we should consider the status of animals as lying on a continuum between domestication and wildness: the more dependent on and adapted to humans an animal is, the more domesticated he/she is and the more positive duties we have toward that animal. The less adapted and dependent, the wilder an animal is. In this case, we have an obligation to protect the animal’s habitat, but we have no duty of care to the specific animal. Regarding ripple effects, it could then be argued that we have more duties toward the lynx and the wolf than toward the rabbit, as the former are more vulnerable as a result of direct human actions, whereas the latter is less vulnerable and the human actions are more indirect.

### 3.1. Species Level

So far, we have looked only at the implications of interventions for individuals of different species. If we were to look at the species level, we could decide that lynxes have more value than rabbits, as there are fewer lynxes left in the wild and the species is at higher risk of going extinct. Moreover, being apex predators, they perform an important role in their ecosystem. From the point of view of species conservation, as long as the species’ population is thriving, we do not need to worry about, for example, the consequences for the European rabbit. In the case of the lynx, on the other hand, doing nothing will most certainly result in the species’ extinction, caused by human actions.

Yet, intervening does not come without a cost: a certain degree of wildness will be lost and this may be another reason to do nothing. Wildness is an important value from an ecocentric perspective, but less so from an animal rights or welfare perspective. Ecocentrism can be described as ‘a worldview that recognizes intrinsic value in ecosystems and the biological and physical elements that they comprise, as well as in the ecological processes that spatially and temporally connect them’ [35] (p. 130). Wildness could be defined as a quality of interactions of organisms and their surroundings, allowing the construction of durable (eco)systems with respect for nature’s autonomy and a lack of human intervention (based on [36,37]). Inspired by the writing of Aldo Leopold [38], ecocentrists tend to value the autonomy, spontaneity, and independence of natural processes and therefore also their wildness [17]. Although wildness is by no means an undisputed category within environmental ethics [39], it does form the basis of many conservation measures. Thinking specifically about animal wildness, Palmer [11] has made a helpful distinction between (1) dispositional wildness, where wild animals do not have a tame character, (2) self-willed wildness, where wild animals are able to do what they want without human controls or constraints, and (3) constitutive wildness, where wild animals are not domesticated, in the sense of being dependent on and adapted to humans, as occurs, for example, in animal breeding. The kind of wildness that is lost when humans intervene depends on the type and level of intervention.

Those who greatly value wildness may argue that we should generally not intervene. On the other hand, wildness is already being reduced by human-induced climate change. The disappearance of species or complete ecosystems may result in even greater reductions in wildness, as this is caused by humans. However, as argued by Palmer [40], climate change is not intended, and the outcomes are not controlled or directed. The way in which nature reacts to this change can still be considered wild. In the case of the lynx, another consideration arises. To what extent can the current lynx population still be considered wild? Will interventions decrease their wildness? In 2002, there were only 94 Iberian lynxes left in the wild, in two fragmented areas [41]. Over time, wild individuals have been held in captivity [42] and reintroduced [43], and additional food has been provided via the translocation of rabbits [44]. Additionally, in areas recently extirpated of lynx, completely new populations were created via a reintroduction program [45]). Many individuals are there thanks to these measures; the population rose to 1111 individuals in 2021 [46].

Dispositional wildness may have been impacted by these measures to some extent, as animals in zoos—and likely also in breeding facilities—become used to the presence of people (i.e., keepers and visitors) and display less fearful and aggressive behavior [47]. Self-willed wildness was temporarily impacted when the lynxes were held in captivity or translocated and may be impacted by human impacts on the ecosystem, but is likely to have returned to its pre-intervention levels. Constitutive wildness, however, is affected by the establishment of new human-made populations via the reintroduction program. From this we can conclude three things. (1) If we argue that the species has already lost most of its overall wildness because of these measures, the wildness of the species might not be impacted greatly by some additional measures. (2) If we argue that, although the species has lost its wildness, the wildness is regained over time, additional measures will result in the loss of wildness in the short term but can also be regained over the long term. (3) If we argue that the species did not lose wildness because of the initial measures, it is likely that additional measures will not result in decreased wildness either. Therefore, for this particular species that is surviving because of interventions, losing wildness is not a valid reason to reject all new interventions. Nevertheless, some measures will result in higher losses of wildness than others. Our interventions could potentially have an impact at the landscape level. Could they make the place where lynxes are living less wild? Or alternatively, could the extinction of the lynx make the place less wild? If we understand the wildness of a landscape as the possibility of having autonomous processes independent of human intervention, then yes, our interventions—such as feeding and making shelters—would make the place less wild. However, as noted, some interventions—breeding and reintroducing lynxes—have already taken place. The effects of the lynx’s extinction on the landscape are not self-evident; autonomous processes would still take place. However, as the extinction is human-induced, wildness in a constitutive sense would still be lost. If we value the survival of the Iberian lynx the most, doing nothing is not morally justified, as this will most definitely result in the species’ extinction. In sum then, both from an individual justice or welfare perspective and from a justice to species perspective, it appears that doing nothing is not morally warranted. We have a duty of care to animals that would be negatively impacted by our interventions, but this duty is less strong than our duty toward the lynx. Moreover, from a species perspective, it appears that an intervention would not in principle make the lynx less wild than previous interventions. To prevent the Iberian lynx from going extinct, human interventions are needed; but what interventions are warranted?

### 3.2. Reducing Other Threats

Besides climate change, other factors threaten the survival of the lynx: the development of urban areas, both timber and non-timber crops, roads and railroads, and hunting and trapping the lynx Animals used to be hunted and trapped for their hide and for trophy hunting, but this is illegal now in Europe. Currently, they die accidentally in fox traps and are killed illegally by hunters because they are regarded as a threat to game populations. (See https://www.thelocal.es/20191004/hunters-kill-four-protected-iberian-lynx-in-central-spain/, accessed 27 January 2023) [48]. Reducing these threats may be one way to prevent the lynx from going extinct as well as decreasing the suffering of individual lynxes; in fact, this may be one of the easiest and least controversial measures that can be applied. As most threats are human-induced, removing these threats does not result in ethical dilemmas such as a decrease in wildness, the suffering of a few individuals to prevent overall suffering, or the risk of unwanted ecosystem modifications. Reducing other human-induced threats can even increase the wildness of the area, as the human impact on the ecosystem will decline. However, reducing these threats may conflict with the interests of humans—for example, rural development. These conflicts can be avoided to a certain extent by, for example, building road underpasses and wildlife corridors to decrease urbanization threats and by better enforcement of the ban on hunting lynxes. Some measures have already been taken, such as the clearing of shrubland, the removal of forestry plantations, and the construction of underpasses under roads [49]. Unfortunately, these measures have been executed insufficiently and only in small areas [33].

The question is whether reducing other threats is enough to prevent the species from going extinct. The fragmentation and destruction of habitats that have already taken place prevent the lynxes from dispersing in long-term suitable habitats. Climate change will, over time, make the current habitats unsuitable for the lynxes’ continued survival. Fordham et al. [22] expect that reducing other threats could delay the extinction of the lynx by less than 15 years. Decreasing other threats until permanent decisions are taken seems like a logical step, so that we at least have extra time and decrease the risk of the Iberian lynx going extinct sooner than expected. Nevertheless, to ensure the Iberian lynx’s survival not only in the short term but also in the long term, additional measures are needed.

## 4. Providing Food and Shelter

One such additional measure is providing food or shelter, or both. As outlined above, when specific measures are taken to benefit individual lynxes, this is likely to have an impact on other animals. Both offering food and providing shelter to the lynx could create unfair competition between the individual lynxes and individuals of other species. Moreover, we can wonder whether it is morally permissible to breed individual animals as feed to prevent other individual animals from dying. Rabbits, if raised for consumption for the lynx, would undergo suffering, a lack of possibility to perform natural behaviors, life in captivity, and loss of life, so that other individuals (the lynxes) would not starve to death. As one Iberian lynx eats many rabbits in his/her lifetime, this measure is expected to increase overall suffering, making this problematic from a utilitarian point of view. Moreover, the suffering that the lynxes would undergo if they were not fed is caused indirectly by humans and the outcome is not intended, whereas suffering because of being raised for consumption is both intentionally and directly caused by humans, potentially making this action unjustifiable from a deontological viewpoint.

Purely at the individual animal level, then, there does not seem to be much moral justification to sacrifice many individuals of one species to prevent the death of fewer individuals of another species. This conclusion may, however, be premature, because it could be argued that wild predators, such as the lynx, need to eat other animals in order to survive. Should we deny lynxes their natural behavior and means of survival? This question relates to a discussion among animal ethicists about the predator problem. Animal ethicists argue that we should not harm animals unnecessarily, but does this not imply that we should also stop animals from harming one another? What was first suggested as a reductio ad absurdum, which was meant to undermine the view that we should help animals, has now been taken up seriously by some animal ethicists. For example, Nussbaum [50] argues that, if we have a duty to stop the suffering of animals that we raise for food, this duty should also extend to helping animals who are suffering in the wild when they are consumed by predators. Some even argue that it would be better if predators went extinct entirely [51]. The view here is that we have a duty to interfere in the wild to alleviate wild animal suffering even if this suffering is not caused by humans and is therefore natural. Others argue that, if we are not responsible for the suffering of prey animals, we do not have an obligation to help them; we cannot blame predators for carrying out their natural behavior, as they are not moral agents, and we should adopt a hands-off approach [12,52]. On the other hand, if the predators are in our care, such as in a zoo, we—at least indirectly—cause the suffering of the prey animals that they eat, and some argue that this does create a moral responsibility on the part of humans for the actions of the predator [53]. Similarly, a moral responsibility could be based on our conservation efforts, when we increase the numbers of prey in an area. More generally, it has been argued that, because of anthropogenic climate change, we are entangled with the lives of wild animals to such an extent that we have at least some moral responsibility to alleviate their suffering [54] (However, this entanglement could mean different things in the case at hand: anthropogenic climate change has caused the predicament in which the lynxes find themselves, and this would point to a duty on our part to feed them. On the other hand, the rabbit population has also decreased as a result of climate change, entailing a human duty to help the rabbits. This, however, may give us an additional reason to breed rabbits, even if many of them will be fed to lynxes, if this simultaneously helps to stabilize the rabbit population).

Others, on the other hand, argue that wild animals are perfectly capable of looking after themselves. Prey animals have evolved unique capabilities for coping with predators; to a large extent, predation pressures even determine the nature of these prey animals [11,55]. In other words, at population or species levels, predation serves an important purpose, even though individual prey animals stand to suffer. In our view, this is not simply a conflict between individualist animal ethicists on the one hand and collectivist ecocentrists on the other. Individual animals are also generally better off when their population is more robust, and this sometimes entails weaker individuals dying so that other individuals will become more robust [56]. An example is given by Cripps [57]: when the American cheetah went extinct in the US, one of its main sources of food, the pronghorn antelope, became less robust. Without the cheetah’s predation pressure, antelopes could no longer flourish fully, because they could not develop a number of important traits, such as speed and visual acuity (see also [11]). In other words, even if justice requires that we help prey animals, this may often still mean that a hands-off approach is the best course of action.

To connect this complex discussion to the case at hand, if we release rabbits into the area so that they can be eaten by lynxes, we may not be morally responsible for preventing the suffering of the rabbits, so long as the rabbits have the opportunity to evolve capabilities to cope with the predation pressure. This realization provides a middle road, so to speak, between a focus solely on the suffering of individuals and a focus solely on the preservation of species—given that the health and wellbeing of most individual animals is served when their population or species is healthy; there is, then, a collective dimension even to the animal ethicist’s approach [56].

### Species Level

If we reason beyond the middle-road perspective suggested above and focus exclusively on the species level, breeding rabbits to prevent the lynx from going extinct also appears justified. Breeding rabbits to feed lynxes does not negatively impact the wild rabbit population and may even result in more wild rabbits if rabbits are released to increase the wild population. If this prevents the lynx from going extinct, one species is saved, without costs to another species. It is not likely that the lynx will dominate the ecosystem and result in the extinction of other species, given its low reproductive capacity and non-invasive characteristics. However, it is important to carry out an impact analysis before such a measure is considered, taking into account the robustness of other animal species and the impact on the overall ecosystem.

Another issue is that feeding or providing shelter may lead to a loss of wildness. It may be necessary to offer food or shelter indefinitely, as the species will go extinct if the aid is stopped, constantly impacting the wildness. This would create a dependency relationship and would likely also impact the wildness of the ecosystem itself or that of other animal species. For example, it may be hard to provide food that will benefit only the lynx itself and not impact other species, as some of the food could be taken by animals of other species. The same applies to providing shelter. There is no guarantee that the shelter provided would be used only by lynxes, potentially decreasing the dispositional wildness or constitutive wildness of other species. Besides impacting wildness, stopping the aid may also lead to the lynxes suffering from starvation. This could be avoided if, rather than just feeding rabbits to the lynxes, the lynxes were trained to hunt the rabbits. This intervention, then, should be considered only if an impact analysis projects that, at a certain juncture, the ecosystem and the predator–prey relationship will achieve a balance so that the aid can be stopped without causing suffering and a permanent loss of wildness; in other words, the goal should be to recreate a situation in which nature can maintain itself. Providing food or shelter should therefore be categorized as a short-term emergency measure to prevent individual lynxes from suffering and to give the species a chance to recover, rather than as a long-term solution.

## 5. Assisted Migration

An intervention that reaches even further is assisted migration or colonization (The difference between assisted migration and colonization is that, for assisted colonization, the lynxes are brought to areas outside of their original, natural dispersal range, whereas, for assisted migration, the lynxes can be relocated to areas where they once lived, but where they are currently extinct. Both may be relevant for the Iberian lynx, as explained in the text). Climate change has made the Iberian Peninsula a less and less suitable habitat for the lynx. To extend the time that they can live in these areas, lynxes can be translocated to other areas within the peninsula with larger surface areas, higher rabbit populations, or better-suited habitats. Nevertheless, increased droughts and the effects on the shrublands will make these areas also unsuitable in due course. It is expected that new, suitable areas in France will arise, with similar habitat characteristics as the areas where the lynxes currently live [21,22]. Unfortunately, the lynxes will not be able to disperse to these areas naturally because of fragmentation of their habitats as a result of urbanization, and therefore researchers have suggested assisted migration [21,22].

Relocation may prevent individuals from dying a cruel death in the short term—for example, from water deficiency or starvation—thereby preventing suffering caused by human-induced climate change. On the other hand, relocation can itself result in increased animal suffering. The process of relocation can cause a lot of stress in some animal species, especially if not executed thoroughly and with respect to individual animal suffering. As many interventions have already been implemented with the Iberian lynx, it is known that individuals can generally withstand and survive sedation and relocation (without long-term consequences). However, cases of convulsions have been reported [58]. Furthermore, different techniques result in differences in the lynx’s ability to cope with the interventions [59]. Soft-release—where individuals are first acclimated in an enclosure prior to release—have been shown to increase activity [60], survival rate, and reproductive rate [61,62,63] compared to hard-releases without an acclimation period. However, in such soft enclosures, stereotypical behavior (i.e., pacing) also occurs [59].

Moreover, the effects on other wild animals should not be ignored. The presence of the lynx in new areas is likely to result in behavioral changes in other animals present, such as anti-predator behavior [64,65]. A ‘landscape of fear’ arises, where other animals (in this case both predators and prey) change their behavior because of the Iberian lynx’s presence (e.g., [66]). One example is that the behavior of foxes has changed since the reintroduction of the lynx in Portugal’s Guadiana valley. Competition with and threats from the lynxes have led the foxes to display more vigilant and aggressive behavior [67]. These behaviors have been linked to higher stress levels and decreased welfare [68,69] and may also result in less suitable foraging areas [70], the adoption of an alternative diet [71], and increased fitness costs [72]. On the other hand, if the lynxes were to be removed from the areas in which they are currently living, changes may also be expected there. It is hard to predict how other top predators, such as the wolf, would react to the lynx’s disappearance. If the wolf does not fill the gap left by the lynx by increasing its population size, the increased suffering that the lynx creates in new habitats may be outweighed by decreased suffering in its old habitat.

### 5.1. Impact on the Ecosystem and Other Species

Fordham et al.’s [22] models show that the annual release of 12 lynxes (aged between 1 and 4 years; 6 of each sex) in habitats classified according to carrying capacity could prevent the extinction of the Iberian lynx this century. Minteer and Collins [73] call this the move it or lose it strategy, where the protection of habitats or the small-scale assistance of some species may not be enough and we have to choose between either relocating species or losing the species. So, assisted migration would be effective in preventing the Iberian lynx from going extinct, but what are the effects of such an impactful measure on the whole ecosystem?

Relocating a species to a new area will have an impact on that recipient area, meaning that we intentionally change ecosystems. Some populations may decrease, while others will increase, new ecological connections will be made, and over time the system will establish a new equilibrium. As Minteer and Collins [73] (p. 1802) argue, ‘Whereas historically we have taken on the role of preservers of species and ecosystems, in the 21st century we will likely find ourselves pressed into a very different role: makers of novel ecosystems for stressed populations, including animal, plant, and human’. There is a broader discussion within conservation biology about the desirability of creating novel ecosystems [74,75] that we have no space to delve into here, save to say that the intentional introduction of the lynx in France may lead to novel ecosystems. However, if we had not created barriers for the lynx in the form of habitat fragmentation and urbanization, a large part of this process would probably have occurred automatically, via dispersal and migration.

Assisted migration or colonization can cause problems for other animals. A major issue when relocating species—and a reason why many ethicists and biologists oppose assisted migration—can be the creation of invasive or pest species. It is not only the species being relocated that can become a pest; diseases and parasites carried by the host can also become invasive [76]. Fortunately, this effect can be minimized by a quarantine period and health checks [77]. The chance of the Iberian lynx causing the extinction of other species cannot be ruled out entirely. However, if we look at the general characteristics of invasive species, the chance of the lynx becoming a pest is quite small (Mueller and Hellmann [78] assessed the characteristics of 468 invasive species in North America. They concluded that intracontinental invasions were far less frequent than intercontinental invasions. Fish and crustaceans especially have a higher risk of becoming invasive within the same continent, whereas plants, invertebrates, and mammals have a lower risk. The severity of the negative effects of the species was, on the other hand, not dependent on whether the species was from the same continent. Invasive mammalian species are often generalists, with a diverse diet, high reproductive rates, and wide habitat tolerance [79,80,81]. These are traits that the Iberian lynx does not possess). Nevertheless, the lynx increases the fitness costs of other species thanks to both competition and the creation of the aforementioned landscape of fear. As both the lynx and many other Iberian predators have the rabbit as their main source of prey [82], increased competition is to be expected.

In the Guadiana valley, the Iberian lynx has been reintroduced since 2015, resulting in a population of more than 100 lynxes in 2019. Research by Sarmento et al. [67] has shown that the red fox populations (*Vulpes vulpes*) have declined drastically in areas where the lynx is present, whereas, in nearby areas where the lynx is absent, fox populations have increased. However, the lynx is not expected to cause the local extinction of these species. Such extinctions would have large impacts and could also result in ecosystem degradations, given the regulating function that the predators have on rodents [83], which in turn could result in population booms of these animals, altering the ecosystem. Therefore, prior to the release of lynxes in new areas, it is necessary to conduct a complete assessment of the ecosystems in order to be as certain as possible that no other species will decline to vulnerable levels. As long as some of the individuals of the predator species move to nearby areas and their presence in the area is not completely lost, the impact that the lynx is expected to have on the other predators does not seem to be a problem from a species conservation and ecosystem point of view.

### 5.2. Wildness and Human–Wildlife Conflicts

Regardless of whether the species will impact the recipient ecosystem positively, assisted migration may decrease that ecosystem’s wildness. It is important to decide whether to relocate the species to areas where the lynx once occurred but has gone extinct or to areas where they are not native. If we base our introduction strategy on whether the area is the most suitable for the lynx and not whether the lynx is native in that area, we can expect a population of 654 to 896 lynxes in 25 to 31 subpopulations [22]. However, if we reintroduce the lynxes only in areas where they are native (but have gone extinct), there will be fewer remaining populations (190 to 275 animals in 7 to 10 subpopulations [22]. Furthermore, to prevent the population from declining after 2065, areas in France, where the lynx is not native, may need to be chosen, as the original range of the lynx will likely become unsuitable for the species to survive [21,22,84].

The relocation is not likely to decrease dispositional and constitutive wildness, except when lynxes are bred in captivity, but self-willed wildness will be decreased, as the animals are not able to do what they want but are ‘forced’ to relocate. On the other hand, the lynxes’ self-willed wildness has already been decreased by current human constraints, such as roads and cities. Therefore, we could argue that the Iberian lynx’s self-willed wildness is already low because of its inability to move, and that relocating the species to areas where it has more potential can actually increase the long-term wildness of the species when there are fewer human controls or constraints in the recipient area—e.g., larger connected habitats—compared with the former habitat.

Another risk is human–wildlife conflicts. For carnivores in general, conflict with humans is the major cause of declines in populations [85]. Native predators are often opposed by locals because of the loss of livestock or the perceived risk for humans [86]. The introduction of an animal perceived as dangerous may result in even worse resistance. When lynxes are relocated to areas in which they have not previously occurred, perceptions about the introduction are very important, as a lack of support could, in a worst-case scenario, result in the death of lynxes and the failure of the relocation process. Given its small size, the Iberian lynx does not generally come into conflict with humans [87]. The species is also no risk for humans, with no reports of attacks by lynxes [88]. However, an increased number of attacks by the Iberian lynx on livestock have been recorded; poultry in particular are targeted, and sheep to a lesser extent [89]. In the habitats where the lynxes live now, the farmers affected by the attacks are compensated.

In short, there is a lot of uncertainty regarding the effect of relocation of the Iberian lynx. It is likely that relocation will result in the survival of the species if human–wildlife conflicts in the recipient area are minimized, for example, by offering farmers compensation. Additionally, the lynx, given its traits, is unlikely to become an invasive species but will likely cause the decline of individuals of other species as a consequence of competition for food and the creation of a landscape of fear. More research is needed to predict whether the lynx will have a net positive or a net negative effect on the recipient ecosystem. As the measure will be executed by humans, the wildness of the ecosystem will decrease; the extent to which this will happen is ambiguous. When the survival of the species is valued most, there are many strong arguments in favor of managed relocation. For this strategy, mitigating climate change is crucial. As Albrecht et al. [90] argue, without adequate climate change mitigation, we expose animal species to both the pain and the distress that result from the effects of climate change as well as from the relocation process. Then, move it or lose it may turn into move it and lose it.

## 6. Bringing Lynx Populations into Captivity

A last-resort measure to prevent the species from going extinct could be bringing Iberian lynx populations into captivity. Captivity entails removing wild animals, populations, or complete species from the wild and bringing them into artificial refuges or unnatural zoo environments or into semi-natural, managed environments. Bringing populations into captivity could, in theory, be used to decrease the suffering of individual Iberian lynxes. This measure reflects a discussion in animal ethics—related to our earlier discussions of wild animal suffering and the predator problem—about the question of whether animals suffer more in the wild or in captivity. Some authors argue that nature is (one of the) worst things that can happen to animals [91]. Johannsen [92] (p. 338), for example, claims that ‘at best, most wild animals born into the wild fail to flourish, and at worst, most do not have lives worth living’. According to Faria [93], ‘Mother Nature is so cruel to her children, she makes Frank Perdue look like a saint’. Frank Perdue (1920–2005) was the president of one of the largest chicken companies of the United States. The animal welfare organisation People for the Ethical Treatment of Animals (PETA) remembers Frank Perdue as ‘the man directly responsible for more animal suffering and deaths than perhaps any human in history’ [94]). Following this point of view, perhaps we should bring as many wild animals into captivity as possible. However, it is disputed whether in the wild animal suffering actually outweighs animal enjoyment [95]. Moreover, it is illogical to compare the unnecessary intentionally caused cruelty that animals in factory farms must undergo with the unintentional suffering of wild animals.

It seems that, in this discussion, proponents and opponents have different understandings of animal welfare Three broad views on animal welfare can be distinguished: function-based, feeling-based, and nature-based views [96]. According to the first, the main question is whether animals can cope with the environmental pressures that they encounter; according to the second, an animal has good welfare if he/she feels good; whereas, according to the third, animals have good welfare if they can display natural behavior. Increasingly, in discussions about welfare, it is emphasized that not only the absence of pain or suffering, but also the presence of positive experiences, is important [97]. These positive experiences are often related to freedom of choice and the opportunity to carry out natural behavior, for example, the possibility to choose one’s own mate and to decide what to eat and to establish social hierarchies. The abovementioned claim that the welfare of wild animals is poorer than that of animals in factory farms appears to rest on a narrow view of function- and feeling-based welfare and ignores welfare views that emphasize positive welfare, in this case, the ability to perform natural behaviors, have social structures, and reproduce naturally. Moreover, following Palmer’s [52] contextual ethics, we have a positive duty to protect the welfare of domesticated animals, but we do not have a moral obligation to protect all wild animals from suffering. Even if we had such an obligation, it is questionable whether large-scale interventions in nature would really increase animal welfare, again depending on one’s view of welfare. Thinking again about the lynx’s food web, it is difficult to predict whether the removal of the lynx will have a net positive effect because of its lower fitness costs for other animals.

In practice, bringing the lynx into captivity in our view is likely to lead to suffering. Especially in artificial areas (zoos), but also in semi-natural closed environments, animals are less able to perform natural behaviors. More diseases are found in captive felid species than in wild individuals [98], and the performance of stereotypical behavior (indicating stress) remains a big problem [99]. In earlier Iberian lynx breeding programs, up to 7.44% of lynx cubs were diagnosed with epilepsy; half of the diagnosed lynxes died during the time of the study [100]. Manteca [99] mentions that captive felids have a higher presence of diseases, difficulty adapting to a challenging (zoo) environment, an inability to express normal behavior (feeding, moving, and exploring), resulting in the (increased) presence of maternal cannibalism, delayed birth, brain hypoxia, anorexia, and aggression. More than half of first-time captive Iberian lynx mothers show maternal neglect [101]. These welfare problems drastically increase the risk that removing the lynxes from the wild will not decrease suffering but will in fact result in increased suffering.

If we look at this issue from the point of view of justice owed to individual animals, two different routes can be taken. On the one hand, rights theorists such as Tom Regan argue that bringing animals into captivity interferes with their right to freedom [12]. Similarly, Donaldson and Kymlicka [55] argue that wild animals have territorial rights on which we should not infringe. On the other hand, some rights theorists argue that we have a duty of beneficence to assist wild animals who suffer and that this may entail bringing them into captivity [102]. Therefore, there is ambiguity around what we should do from a justice perspective.

### Effects on the Species

When the Iberian lynx is brought into captivity in different, artificial populations, it can be questioned what values are preserved. It seems that the main value is anthropocentric, namely, that our children will still be able to enjoy seeing the species, albeit in captivity. Conserving species is important among other things because of the role they play in ecosystems, but this role vanishes when species are taken out of their natural habitats and placed in zoos. Captivity may furthermore lead to a loss in self-willed and dispositional wildness, as the animals are unable to perform all their natural behaviors, become used to human presence, and become stressed, leading to unnatural behaviors.

The impact on constitutive wildness raises even more questions. To what extent does the Iberian lynx remain the Iberian lynx when it is bred—in small numbers—in captivity? A lot of research warns about the effects of a lack of genetic diversity or unintended selection in captive breeding programs [103,104,105]. Human-induced changes may happen unintentionally. The least tame lynxes may experience more stress, have shorter lives, and have fewer offspring than the lynxes that are less affected by the captive settings, leading to a process of domestication. One unwanted effect of unintended selection that is often seen in wild animal breeding programs is a decline in the species’ fitness [106]. The longer individuals are kept in zoos, the more difficult it is for them to survive in the wild. When the aim is to keep the Iberian lynxes in captivity forever, instead of in the wild, it can be questioned whether over long periods of time the Iberian lynx remains the Iberian lynx and is not significantly altered by unintended selection or a lack of genetic diversity. Additionally, when not all lynxes are removed from the wild, but only a few individuals, this is likely to result in the drastic decline of the wild population. After all, the genetic diversity of the wild population is already very poor, and the population is very vulnerable. Removing additional individuals increases the rate of population collapse and would therefore not be a viable solution to save the species.

## 7. Discussion and Conclusions

We have discussed the merits of five intervention strategies for helping the Iberian lynx cope with the effects of climate change, from the point of view both of the welfare of, and justice to, individual lynxes and of species conservation. Table 1 gives an overview of the discussed interventions and their expected effects on the individual welfare of lynxes and other animals affected by this measure, as well as the effects on the conservation of the Iberian lynx and other affected species. For some interventions, outcomes can be either positive or negative (or both). For example, doing nothing will be negative for the welfare of Iberian lynxes; however, because of this, it may turn out to be beneficial for the welfare of prey animals. Assisted migration will likely be beneficial for the welfare of the lynxes but may be negative for the welfare of prey animals in the recipient area. Still, this effect may be outweighed by the positive effect that the removal of the lynxes will have on the welfare of prey species in the old area. We have not included the viewpoint of justice to individual lynxes in the table, because the table is about positive and negative effects and this does not fit easily with the justice viewpoint. From a justice viewpoint, doing nothing is problematic, as we have caused the lynxes’ predicament. Reducing other threats is preferable from a justice point of view. Providing food is problematic for the rabbits, but we may have a duty toward the lynxes to do so (depending on how one deals with the predator problem). Captivity is problematic if we argue that (wild) animals have a right to freedom. Assisted migration is ambiguous, because the lynxes have a right to non-interference, but, at the same time, they potentially regain freedom after the migration.

Reducing other threats, such as roads and hunting, is the least controversial measure from both an animal welfare and a species conservation point of view but will not be enough to prevent the Iberian lynx from going extinct. Therefore, additional measures are needed. Providing food or shelter is problematic from both an animal welfare point of view and a species conservation point of view. If we were to provide food (i.e., rabbits) to prevent the Iberian lynx suffering, we would increase overall suffering, as rabbits need to be bred, kept in captivity, and killed for this measure. This measure could be used to prevent the Iberian lynx from going extinct in the short term but may give us a permanent obligation, as ceasing this intervention may have the species’ extinction as a consequence. Depending on how exactly feeding is carried out, and, for example, whether the lynxes are trained to hunt for their own food, this measure risks decreasing the wildness of the species and the ecosystem. Bringing populations into captivity in our view is not justified; the animals will likely suffer more in captivity than in the wild and it is questionable which values are kept intact by doing this. Although more research is needed and uncertainties need to be resolved before possible implementation, assisted migration may, among other things because of the Iberian lynx’s non-invasive characteristics, be the best way to protect the species while ensuring animal welfare and protecting wildness and other ecosystem values. When implementing assisted migration, we should aim for the soft-release strategy, place the lynxes in quarantine, and perform health checks. Moreover, an impact assessment for the recipient ecosystem needs to be carried out.

To decide whether measures should be undertaken to conserve species and/or to increase the welfare of individual animals, a case-by-case approach is crucial. Specific conclusions drawn in this paper can be applied only to the Iberian lynx and maybe a few other species, with similar characteristics and circumstances, but cannot be extrapolated to all species. We discussed, for example, how, without human influence in the past, the Iberian lynx might have been able to move toward new suitable areas in France, whereby relocating toward these areas would have impacted wildness less than one would expect and potentially even increase wildness. This is a totally different scenario than that discussed by Albrecht et al. [90], who explored the possibility of relocating the polar bear to the Antarctic. Without human influence, the polar bear will not be able to move from the Arctic to the Antarctic. Therefore, relocating this species will impact the wildness substantially, in contrast to the case of the Iberian lynx.

Likewise, Palmer [40] researched the American pika. In contrast to our research, she concludes that assisted migration will probably not be supported if we most value the protection of ecosystems, the protection of wildness, or animal rights. However, this difference can also be explained to some extent by the history of interventions resulting in the current Iberian lynx populations, the inability of pika to migrate naturally, in contrast to the Iberian lynx, and the process of relocation being more stressful for pikas than for Iberian lynxes.

Although the specifics of our case study are not generalizable to other cases, certain insights and lines of reasoning may very well be. In our discussion, we touched on a number of broader discussions taking place in animal ethics, such as those about the predator problem, wild animal suffering, and the conflict between individualist animal ethics and collectivist conservation ethics. Regarding the latter, we hope to have shown that, in practice, it is very possible to take both perspectives into account. For example, it is often argued that bringing animals into captivity is problematic from an individual animal welfare point of view, but not from a conservationist point of view. We have argued that, from a conservationists’ point of view also, it is doubtful that this is a good measure, as it interferes with the value of wildness that is central to the conservationist perspective and it does not aid in the protection of ecosystems.

Additionally, at first sight, feeding lynxes with specially bred rabbits seems problematic only from an animal ethics point of view, given that, for many animal ethicists, the interests of each individual should be weighed equally and this measure sacrifices many rabbits in order to save a small number of lynxes. On second thoughts, however, feeding also seems problematic from a conservation perspective, as it may lead to a disturbance of the food web and the wider ecosystem and to a decrease in wildness. Moreover, our discussion of the predator problem showed that even if we attribute moral status to individual animals only, we have good reason to take the species dimension into account. After all, it is in the interest of the individuals comprising the species that their species (or population) is robust and healthy, and this may mean that individual interests sometimes need to be sacrificed (see also [56] for a broader discussion of this point). Therefore, if we use a case-by-case approach, the underlying theoretical conflict between animal ethics and conservation may not fully disappear, but it can often be bridged in practice. Justice demands that we find ways of helping animals affected by climate change and that we do so by being attentive to the particular contexts in which these animals find themselves.

## Figures and Tables

**Table 1:** Overview of the measures and the likely effects on individual animal welfare and species conservation.

	Doing Nothing	Reducing other Threats	Providing Food or Shelter	Assisted Migration/Colonization	Bringing Populations into Captivity
Individual animal welfare	+/−	++	−−	+/−	−−
Species conservation	−−	+	−	++	+/−

Note: ++ is the most positive expected effect, followed by +. −− is the worst expected effect, followed by −.

## Data Availability

Data is contained within the article. This article is based on literature research only.

## References

[1] Masson-Delmotte V., Zhai P., Pirani A., Connors S.L., Péan C., Berger S., Caud N., Chen Y., Goldfarb L., Gomis M.I., IPCC (2021). Climate Change 2021: The Physical Science Basis. Contribution of Working Group I to the Sixth Assessment Report of the Intergovernmental Panel on Climate Change.

[2] Pielke R., Burgess M.G., Ritchie J. (2021). Most Plausible 2005-2040 Emissions Scenarios Project Less Than 2.5 Degrees C of Warming by 2100.

[3] Brondízio E.S., Settele J., Díaz S., Ngo H.T., IPBES (2019). Global Assessment Report of the Intergovernmental Science-Policy Platform on Biodiversity and Ecosystem Services.

[4] Urban M.C. (2015). Accelerating extinction risk from climate change. Science.

[5] Shue H. (2014). Climate Justice: Vulnerability and Protection.

[6] Pepper A. (2018). Adapting to climate change: What we owe to other animals. J. Appl. Philos..

[7] Tschakert P., Schlosberg D., Celermajer D., Rickards L., Winter C., Thaler M., Stewart-Harawira M., Verlie B. (2021). Multispecies justice: Climate just futures with, for and beyond humans. WIREs Clim. Change.

[8] Blattner C.E., Meijer E., Schübel H., Wallimann-Helmer I. (2021). Animals and climate change. Justice and Food Security in a Changing Climate.

[9] Swart J.A.A., Keulartz J. (2011). Wild animals in our backyard. A contextual approach to the intrinsic value of animals. Acta Biotheor..

[10] Palmer C., Bovenkerk B., Keulartz J. (2016). Climate change, ethics and the wildness of wild animals. Animal Ethics in the Age of Humans: Blurring Boundaries in Human-animal Relationships.

[11] Palmer C. (2016). Saving species but losing wildness? Should we genetically adapt wild animal species to help them respond to climate change?. Midwest Stud. Philos..

[12] Regan T. (2004). The Case for Animal Rights.

[13] Singer P. (1975). Animal Liberation.

[14] Keulartz J. (2016). Should the lion eat straw like the ox?. Animal ethics and the predation problem. J. Agric. Environ. Ethics.

[15] Singer P. (1999). Practical Ethics.

[16] Regan T., Norton B., Hutchins M., Maple T., Stevens E. (1995). Are zoos morally defensible. Ethics on the Ark: Zoos, Animal Welfare, and Wildlife Conservation.

[17] Rolston III H., Callicott J.B., Nelson M.P. (1998). The wilderness idea reaffirmed. The Great New Wilderness Debate. An Expansive Collection of Writings Defining Wilderness from John Muir to Gary Snyder.

[18] Varner G., Light A., Rolston H., III (2003). Can animal rights activists be environmentalists?. Environmental Ethics. An Anthology.

[19] Horta O. (2017). Animal suffering in nature. Environ. Ethics.

[20] Milburn J. (2021). Welcoming, wild animals, and obligations to assist. J. Agric. Environ. Ethics.

[21] Kıraç A. (2021). Potential distribution of two lynx species in Europe under palaeoclimatological scenarios and anthropogenic climate change scenarios. CERNE.

[22] Fordham D.A., Akçakaya H.R., Brook B.W., Rodríguez A., Alves P.C., Civantos E., Triviño M., Watts M.J., Araújo M.B. (2013). Adapted conservation measures are required to save the Iberian lynx in a changing climate. Nat. Clim. Change.

[23] Miranda J.D., Armas C., Padilla F.M., Pugnaire F.I. (2011). Climatic change and rainfall patterns: Effects on semi-arid plant communities of the Iberian Southeast. J. Arid. Environ..

[24] Rodríguez R., Ramírez O., Valdiosera C.E., García N., Alda F., Madurell-Malapeira J., Marmi J., Doadrio I., Willerslev E., Götherström A. (2011). 50,000 years of genetic uniformity in the critically endangered Iberian lynx. Mol. Ecol..

[25] Alfaya P., Casanovas J.G., Lobón-Rovira J., Matallanas B., Cruz A., Arana P., Alonso G. (2019). Using MaxEnt algorithm to assess habitat suitability of a potential Iberian lynx population in central Iberian Peninsula. Community Ecol..

[26] Alda F., Inogés J., Alcaraz L., Oria J., Aranda A., Doadrio I. (2008). Looking for the Iberian lynx in central Spain: A needle in a haystack?. Anim. Conserv..

[27] Minteer B.A., Collins J.P. (2012). Species conservation, rapid environmental change, and ecological ethics. Nat. Educ. Knowl..

[28] Delon N., Purves D. (2018). Wild animal suffering is intractable. J. Agric. Environ. Ethics.

[29] Palmer C. (2018). Should we offer assistance to both wild and domesticated animals?. Harv. Rev. Philos..

[30] Boitani L., Phillips M., Jhala Y. (2018). "Canis Lupus". IUCN Red List of Threatened Species 2020, Errata Version of 2018 Assessment.

[31] Yamaguchi N., Kitchener A., Driscoll C., Nussberger B. (2015). Felis Silvestris. The IUCN Red List of Threatened Species 2015.

[32] Apostolico F., Vercillo F., La Porta G., Ragni B. (2015). Long-term changes in diet and trophic niche of the European wildcat (Felis silvestris silvestris) in Italy. Mammal Res..

[33] Palomares F. (1993). Opportunistic feeding of the Egyptian mongoose, *Herpertes ichneumon* (L.) in Southwestern Spain. Rev. D’écologie (La Terre Et La Vie).

[34] Fedriani J.M., Palomares F., Delibes M. (1999). Niche relations among three sympatric Mediterranean carnivores. Oecologia.

[35] Gray J., Whyte I., Curry P. (2018). Ecocentrism: What it means and what it implies. Ecol. Citiz..

[36] Cookson L.J. (2011). A definition for wildness. Ecopsychology.

[37] Ridder B. (2007). The naturalness versus wildness debate: Ambiguity, inconsistency, and unattainable objectivity. Restor. Ecol..

[38] Leopold A. (1949). A Sand County Almanac: And Sketches Here and There.

[39] Callicott J.B., Callicott J.B., Nelson M.P. (1998). The wilderness idea revisited: The sustainable development alternative. The Great New Wilderness Debate. An Expansive Collection of Writings Defining Wilderness from John Muir to Gary Snyder.

[40] Palmer C. (2018). Conservation strategies in a changing climate–moving beyond an ‘animal liberation/environmental ethics’ divide. Les Ateliers De L’éthique/Ethics Forum.

[41] Lopez G., Lopez M., Fernandez L., Ruiz G., Arenas R., Del Rey T., Gil J.M., Garrote G., Garcia M., Simon M. (2012). Population development of the Iberian lynx since 2002. Cat News.

[42] Meli M.L., Cattori V., Martínez F., López G., Vargas A., Simón M.A., Zorrilla I., Muñoz A., Palomares F., López-Bao J. (2009). Feline leukemia virus and other pathogens as important threats to the survival of the critically endangered Iberian lynx (Lynx pardinus). Public Libr. Sci. ONE.

[43] Palomares F., Rodríguez A., Revilla E., López-Bao J.V., Calzada J. (2010). Assessment of the conservation efforts to prevent extinction of the Iberian lynx. Conserv. Biol..

[44] Fernández N. (2005). Spatial patterns in European rabbit abundance after a population collapse. Landsc. Ecol..

[45] Simón M.A., Gil-Sánchez J.M., Ruiz G., Garrote G., Mccain E.B., Fernández L., López-Parra M., Rojas E., Arenas-Rojas R., Del Rey T. (2012). Reverse of the decline of the endangered Iberian lynx. Conserv. Biol..

[46] Lusa Pela primeira vez em 20 anos, há mais de 1000 linces-ibéricos. Público. https://www.publico.pt/2021/05/28/p3/noticia/primeira-20-anos-ha-1000-lincesibericos-1964416.

[47] Martin R.A., Melfi V. (2016). A comparison of zoo animal behavior in the presence of ffamiliar and unfamiliar people. J. Appl. Anim. Welf. Sci..

[48] Rodríguez A., Calzada J. (2015). Lynx pardinus. The IUCN Red List of Threatened Species 2015.

[49] Ferreras P., Rodríguez A., Palomares F., Delibes M., Macdonald D.W., Loveridge J.A. (2010). Iberian lynx: The uncertain future of a critically endangered cat. Biology and Conservation of Wild Felids.

[50] Nussbaum M.C. (2006). Frontiers of Justice. Disability, Nationality, Species Membership.

[51] McMahan J. The meat eaters. The New York Times 2010. http://opinionator.blogs.nytimes.com/2010/09/19/the-meat-eaters/.

[52] Palmer C. (2010). Animal Ethics in Context.

[53] Milburn J. (2015). Rabbits, stoats and the predator problem: Why a strong animal rights position need not call for human intervention to protect prey from predators. Res. Publica.

[54] Milburn J. (2022). Just Fodder. The Ethics of Feeding Animals.

[55] Donaldson S., Kymlicka W. (2011). Zoopolis. A Political Theory of Animal Rights.

[56] Bovenkerk B., Verweij M., Bovenkerk B., Keulartz J. (2016). Between individualistic animal ethics and holistic environmental ethics. Blurring the boundaries. Animal Ethics in the Age of Humans. Blurring Boundaries in Human-Animal Relationships.

[57] Cripps E. (2010). Saving the polar bear, saving the world: Can the capabilities approach do justice to humans, animals and ecosystems?. Res. Publica.

[58] Ferreras P., Aldama J.J., Beltrán J.F., Delibes M. (1994). Immobilization of the endangered Iberian lynx with xylazine- and ketamine-hydrochloride. J. Wildl. Dis..

[59] Figueiredo A., Torres R.T., Pratas-Santiage L.P., Pérez S., Fonseca C., González M.J.P., Nájera F. (2019). Reintroduction of the Iberian lynx (Lynx pardinus): A preliminary case study in Extremadura, Spain. J. Ethol..

[60] Devineau O., Shenk T.M., Doherty P.F., White G.C., Kahn R.H. (2011). Assessing release protocols for Canada lynx reintroduction in Colorado. J. Wildl. Manag..

[61] Mitchell A.M., Wellicome T.I., Brodie D., Cheng K.M. (2011). Captive-reared burrowing owls show higher site-affinity, survival, and reproductive performance when reintroduced using a soft-release. Biol. Conserv..

[62] Wanless R.M., Cunningham J., Hockey P.A., Wanless J., White R.W., Wiseman R. (2002). The success of a soft-release reintroduction of the flightless Aldabra rail (Dryolimnas [cuvieri] aldabranus) on Aldabra Atoll, Seychelles. Biol. Conserv..

[63] Teixeira C.P., De Azevedo C.S., Mendl M., Cipreste C.F., Young R.J. (2007). Revisiting translocation and reintroduction programmes: The importance of considering stress. Anim. Behav..

[64] Haswell P.M., Kusak J., Hayward M.W. (2017). Large carnivore impacts are context–dependent. Food Webs.

[65] Lima S.L. (1998). Nonlethal effects in the ecology of predator–prey interactions. BioScience.

[66] Laundre J.W., Hernandez L., Ripple W.J. (2010). The landscape of fear: Ecological implications of being afraid. Open Ecol. J..

[67] Sarmento P., Bandeira V., Gomes P., Carrapato C., Eira C., Fonseca C. (2021). Adapt or perish: How the Iberian lynx reintroduction affects fox abundance and behaviour. Hystrix.

[68] Voellmy I.K., Goncalves I.B., Barrette M., Monfort S.L., Manser M.B. (2014). Mean fecal glucocorticoid metabolites are associated with vigilance, whereas immediate cortisol levels better reflect acute anti-predator responses in meerkats. Horm. Behav..

[69] Takahashi A., Flanigan M.E., McEwen B.S., Russo S.J. (2018). Aggression, social stress, and the immune system in humans and animal models. Front. Behav. Neurosci..

[70] Ritchie E.G., Johnson C.N. (2009). Predator interactions, mesopredator release and biodiversity conservation. Ecol. Lett..

[71] Durant S.M. (2000). Living with the enemy: Avoidance of hyenas and lions by cheetahs in the Serengeti. Behav. Ecol..

[72] Creel S., Christianson D. (2008). Relationships between direct predation and risk effects. Trends Ecol. Evol..

[73] Minteer B.A., Collins J.P. (2010). Move it or lose it? The ecological ethics of relocating species under climate change. Ecol. Appl..

[74] Marris E. (2009). Ragamuffin Earth. Nature.

[75] Hobbs R.J., Higgs E., Harris J.A. (2009). Novel ecosystems: Implications for conservation and restoration. Trends Ecol. Evol..

[76] Hoegh-Guldberg O., Hughes L., McIntyre S., Lindenmayer D.B., Parmesan C., Possingham H.P., Thomas C.D. (2008). Assisted colonization and rapid climate change. Science.

[77] Wilkening J.L., Ray C., Ramsay N., Klingler K. (2015). Alpine biodiversity and assisted migration: The case of the American pika (*Ochotona princeps*). Biodiversity.

[78] Mueller J., Hellman J. (2008). An assessment of invasion risk from assisted colonization. Conserv. Biol..

[79] Long J.L. (2003). Introduced Mammals of the World, Their History, Distribution and Influence.

[80] Clout M.N., Russel J.C. (2007). The invasion ecology of mammals: A global perspective. Wildl. Res..

[81] Da Rosa C.A., Zenni R., Ziller S.R., De Almeida Curi N., Passamani M. (2018). Assessing the risk of invasion of species in the pet trade in Brazil. Perspect. Ecol. Conserv..

[82] Virgós E., Lozano J., Cabezas–Díaz S., Mangas J.G. (2011). The presence of a “competitor pit effect” compromises wild rabbit (*Orcytolagus cuniculus*) conservation. Anim. Biol..

[83] Egle S., Davison J., Süld K., Valdmann H., Laurimaa L., Saarma U. (2017). Europewide biogeographical patterns in the diet of an ecologically and epidemiologically important mesopredator, the red fox Vulpes vulpes: A quantitative review. Mammal Rev..

[84] Rodríguez A., Delibes M. (2002). Internal structure and patterns of contraction in the geographic range of the Iberian lynx. Ecography.

[85] LeFlore E.G., Fuller T.K., Tomeletso M., Stein A.B. (2019). Livestock depredation by large carnivores in norther Botswana. Glob. Ecol. Conserv..

[86] Van Eeden L.M., Eklund A., Miller J.R.B., Lopez J.V., Chapron G., Cejtin M.R., Crowther M.S., Dickman C.R., Frank J., Krofel M. (2018). Carnivore conservation needs evidence-based livestock protection. PLoS Biol..

[87] Inskip C., Zimmermann A. (2009). Human-felid conflict: A review of patterns and priorities worldwide. Oryx.

[88] Wilson C.J. (2004). Could we live with reintroduced large carnivores in the UK?. Mammal Rev..

[89] Garrote G., López G., Gil-Sánchez J.M., Rojas E., Ruiz M., Bueno J.F., De Lillo S., Rodriguez-Siles J., Martín J.M., Pérez J. (2013). Human–felid conflict as a further handicap to the conservation of the critically endangered Iberian lynx. Eur. J. Wildl. Res..

[90] Albrecht G.A., Brooke C., Bennett D.H., Garnett S.T. (2013). The ethics of assisted colonization in the age of anthropogenic climate change. J. Agric. Environ. Ethics.

[91] Sagoff M. (1984). Animal liberation and environmental ethics: Bad marriage, quick divorce. Osgoode Hall Law J..

[92] Johannsen K. (2017). Animal rights and the problem of r-strategists. Ethical Theory Moral Pract..

[93] Faria C. (2016). Animal ethics goes wild: The problem of wild animal suffering and intervention in nature. Doctoral Thesis.

[94] (2014). PETA. Frank Perdue’s legacy of animal abuse.

[95] Groff Z., Ng Y. (2019). Does suffering dominate enjoyment in the animal kingdom?. An update to welfare biology. Biol. Philos..

[96] Fraser D. (2003). Assessing animal welfare at the farm and group level: The interplay of science and values. Anim. Welf..

[97] Ohl F., van der Staaij F.J. (2012). Animal welfare: At the interface between science and society. Vet. J..

[98] Munson L., Terio K.A., Worley M., Jago M., Bagot-Smith A., Marker L. (2005). Extrinsic factors significantly affect patterns of disease in free-ranging and captive cheetah (Acinonyx jubatus) populations. J. Wildl. Dis..

[99] Manteca X. (2009). Behavioral Problems of Wild Felids in Captivity in Iberian Lynx Ex Situ Conservation: An Interdisciplinary Approach. Fundación Biodiversidad in Collaboration with IUCN Cat Specialist Group. https://dro.deakin.edu.au/eserv/DU:30059179/fanson-reproductivephysiology-evid-2009.pdf#page=144.

[100] Mínguez J.J., El Bouyafrouri Y., Godoy J.A., Rivas A., Fernández J., Asensio V., Serra R., Perez-Aspa M.J., Lorenzo V. (2021). Benign juvenile idiopathic epilepsy in captive Iberian lynx (Lynx pardinus) in the ex situ conservation program (2005–2019). BMC Vet. Res..

[101] Vargas A., Sánchez I., Martínez F., Rivas A., Godoy J.A., Roldan E., Simón M.A., Serra R., Pérez M.J., Sliwa A. (2009). Interdisciplinary Methods in the Iberian Lynx (Lynx pardinus) Conservation Breeding Programme in Iberian Lynx Ex Situ Conservation: An Interdisciplinary Approach. Fundación Biodiversidad in Collaboration with IUCN Cat Specialist Group. https://dro.deakin.edu.au/eserv/DU:30059179/fanson-reproductivephysiology-evid-2009.pdf#page=144.

[102] Johannsen K. (2021). Wild Animal Ethics. The Moral and Political Problem of Wild Animal Suffering.

[103] Philippart J.C. (1995). Is captive breeding an effective solution for the preservation of endemic species?. Biol. Conserv..

[104] Witzenberger K.A., Hochkirch A. (2011). Ex situ conservation genetics: A review of molecular studies on the genetic consequences of captive breeding programmes for endangered animal species. Biodivers. Conserv..

[105] Fraser D. (2008). How well can captive breeding programs conserve biodiversity? A review of salmonids. Evol. Appl..

[106] Baskett M.L., Waples R.S. (2012). Evaluating alternative strategies for minimizing unintended fitness consequences of cultured individuals on wild populations. Conserv. Biol..

